# Novel *Chlamydia* species isolated from snakes are temperature-sensitive and exhibit decreased susceptibility to azithromycin

**DOI:** 10.1038/s41598-018-23897-z

**Published:** 2018-04-04

**Authors:** Eveline Staub, Hanna Marti, Roberta Biondi, Aurora Levi, Manuela Donati, Cory Ann Leonard, Serej D. Ley, Trestan Pillonel, Gilbert Greub, Helena M. B. Seth-Smith, Nicole Borel

**Affiliations:** 1 Institute for Veterinary Pathology, Vetsuisse Faculty, University of Zurich, Winterthurerstrasse 268, CH-8057 Zurich, Switzerland; 2 DIMES, Microbiology, Policlinico S. Orsola, University of Bologna, 40138 Bologna, Italy; 3 Institute of Microbiology, University of Lausanne, Lausanne, CH-1011 Switzerland; 4 Applied Microbiology Research, Department of Biomedicine, University of Basel, Basel, Switzerland; 5 Present Address: SA MRC Centre for Tuberculosis Research, DST/NRF Centre of Excellence for Biomedical Tuberculosis Research, Division of Molecular Biology and Human Genetics, Stellenbosch University, Stellenbosch, South Africa; 6 Present Address: Clinical Microbiology, University Hospital Basel, Basel, Switzerland

**Keywords:** Bacterial pathogenesis, Bacteriology, Infection

## Abstract

*Chlamydia* species have recently been recognized as emerging pathogens in snakes. However, isolation of novel snake chlamydiae is critical and their growth characteristics are largely unknown. In this study, two novel chlamydial species are described: *Chlamydia serpentis* and *Chlamydia poikilothermis*, isolated after attempts on 23 cloacal and choanal swabs from 18 PCR-positive captive snakes originating from different Swiss snake collections. Isolation success, growth curve and infectivity rates over a 48-hour time period were dependent on temperature (37 °C for *C*. *serpentis*, 28 °C for *C*. *poikilothermis*). *C*. *serpentis* and *C*. *poikilothermis* were sensitive to tetracycline and moxifloxacin during evaluation by *in vitro* antibiotic susceptibility assay but intermediate to resistant (2–4 μg/ml) to azithromycin. Whole genome sequencing of the isolates provided proof of the novel species status, and gives insights into the evolution of these branches of genus *Chlamydia*.

## Introduction

Bacteria within the order *Chlamydiales* are biologically unique, obligate intracellular pathogens affecting humans, wild and domesticated mammals and reptiles, comprising to date nine families (reviewed in)^1^. Diseases and complications caused can include late abortions, infertility, trachoma and pneumonia. Chlamydiosis has been detected in both free-ranging and captive reptiles, causing granulomatous lesions in inner organs^2–5^, proliferative pneumonia^6,7^, necrotizing enteritis or myocarditis^8^, hepatitis and conjunctivitis^9,10^ or remaining asymptomatic^7,11,12^. *Chlamydia pneumoniae*, which was initially described as a human respiratory pathogen, is the most commonly reported etiological agent in reptilian Chlamydiosis^13^.

Recent reports indicate that snakes may harbour a significant level of diverse and uncharacterised novel chlamydial species, in addition to *Chlamydia*-like organisms (CLOs)^5^. Through near-full length chlamydial 16S rRNA gene sequencing from 21 choanal or cloacal swab samples, *C*. *pneumoniae* strains were identified as well as seven novel 16S rRNA genotypes, giving the first indication of the presence of potentially novel *Chlamydia* species in snakes^11^. Culture-independent full genome sequencing of genomic DNA obtained from the choana of a captive, clinically inapparent Madagascar tree boa (*Sanzinia madagascariensis volontany*) revealed the novel *Candidatus* species Chlamydia sanzinia^14^ and similarly *Candidatus* Chlamydia corallus from the choana of a captive Amazon basin emerald tree boa (*Corallus batesii*)^15^. These two new genomes show that both species fall in a phylogenetic clade with *C*. *pneumoniae* and *C*. *pecorum*. However, the availability of sample material and the lack of suited *in vitro* culture systems are challenges to further characterization of novel chlamydial strains.

This study used *in vitro* culture of snake cloacal and choanal swabs with eukaryotic host cells at two temperatures (28 °C and 37 °C, considering the variable body temperatures of poikilothermic snake hosts) to isolate four strains belonging to two new chlamydial species: *Chlamydia serpentis* and *Chlamydia poikilothermis*. We have characterised these both phenotypically and through whole genome sequencing (WGS) and present the 12th and 13th species within the family *Chlamydiaceae*.

## Results

### Detection and isolation of chlamydiae in captive snakes

By real-time PCR, 23 swab samples from 18 individual snakes were positive for *Chlamydiaceae*. Five snakes were positive at both sites tested (choana, cloaca) whereas nine snakes had positive results only from the choana and the remaining four only from the cloaca. The *Chlamydiaceae*-positive snakes belonged to the families *Boidae* (n = 6), *Colubridae* (n = 2), *Pythonidae* (n = 4) and *Viperidae* (n = 6). The majority of the snakes (n = 16) were clinically asymptomatic and sampled alive, whereas three samples were taken from two dead snakes at necropsy. Species-specification with the Arraymate microarray revealed the presence of *C*. *pneumoniae* in most samples (n = 16; 69.6%), of which one sample was also positive for *C*. *muridarum*. Five samples could not be further classified (21.7%), of which four were positive for *Chlamydia* sp. In addition, one sample contained *C*. *caviae* and another *C*. *abortus*. Details of *Chlamydiaceae*-positive snakes are given in Table 1.

**Table 1:** Details of snakes positive by real-time PCR for *Chlamydiaceae*. Samples were collected from asymptomatic alive snakes or taken at necropsy from dead snakes.

CollectionID	Snake no.	Sample no.^1^/Strain ID	Snake species	Live (L) Necropsy (N)	Real-time PCR (∅ Ct value)	Real-time PCR (∅ quantity, copies/µl)	Arraymate	Isolation
**A**	**1**	H15-1957-3C	*Pantherophis guttatus*	L	24.9	42′511	*C*. *pneumoniae*	Yes
	**2**	H15-1957-10C H15-1957-10K	*Atheris squamigera*	L	32.5 33.4	330 188	*C*. *pneumonia* *C*. *pneumoniae*	Yes No
**B**	**3**	H15-1971-20C H15-1971-20K	Sanzinia *madagascariensis voluntar*	L	26.0 30.4	21′767 1′280	*Chlamydia*^4^ *Chlamydia*^4^	No Not done
	**4**	H15-1971-22C H15-1971-22K	*Python regius*	L	29.3^2^ 29.7	3′410^2^ 2′105	*C*. *pneumonia* *C*. *pneumoniae*	No No
**C**	**5**	H15-2055-65K	*Python m*. *molurus*	L	18.4/UT^3^	2′354′763/UT^3^	*C*. *pneumoniae*	Not done
	**6**	H15-2055-69C	*Crotalus basilicus*	L	29.5	3′182	*C*. *pneumoniae*	Not done
	**7**	H15-2055-70C	*Crotalus basilicus*	L	26.9	15′388	*C*. *pneumoniae*	Not done
	**8**	H15-2055-71C	*Crotalus basilicus*	L	25.4	36′997	*C*. *pneumoniae*^4^	Not done
	**9**	H15-2055-72C	*Crotalus basilicus*	L	29.2	4′206	*C*. *pneumonia C*. *muridarum*	Not done
	**10**	H15-2055-79C H15-2055-79K	*Python regius*	L	33.7 26.2	190 26′165	*C*. *pneumoniae*^4^ *C*. *pneumoniae*	Not done Not done
**D**	**11**	H15-2833-20C	*Python regius*	L	29.5	2′547	*C*. *pneumoniae*^4^	Not done
**E**	**12**	H15-2898-1C	*Eunectes murinus*	L	30.9	932	*Chlamydia*^4^	No
	**13**	H15-2898-2C	*Eunectes murinus*	L	26.5	16′134	*C*. *pneumoniae*^4^	No
	**14**	H15-2898-3K	*Eunectes murinus*	L	14.1	212′659′086	Negative^5^	No
	**15**	H15-2898-13C	*Eunectes murinus*	L	29.6	9′245	*Chlamydia*^4^	No
**F**	**16**	H15-2641-14K	*Boa constrictor*	L	26.8	17′286	*C*. *abortus*	No
**G**	**17**	S15-212-K	*Crotalus cerastes*	N	24.8	35′424	*C*. *pneumoniae*^4^	Not done
**H**	**18**	S15-834C S15-834K	*Pantherophis guttatus*	N	25.0 18.2	14′272 4′372′828	*C*. *caviae* *C*. *pneumoniae*	Yes Yes

^1^C = swab samples from choana; K = swab sample from cloaca.

^2^Samples diluted 1:10 for analysis.

^3^UT = undetermined.

^4^Suspected genus/species based on Arraymate probes.

^5^Possible false-positive real-time PCR result.

Isolation was attempted on swab samples (n = 13) from ten of the *Chlamydiaceae*-positive snakes. Of these, isolation of *Chlamydia* at 28 °C or 37 °C was successful in four samples from three snakes belonging to the species *Pantheropis guttatus* and *Atheris squamigera*. Contamination with predominantly gram-negative, rod-shaped but unclassified bacteria hampered the isolation process and overgrew any *Chlamydia* in the rest of the samples. Isolation was successful at 28 °C for the strains named H15-1957-3C, at 37 °C for H15-1957-10C, and at both temperatures for S15-834C and S15-834K.

### Phenotypic characterization of field isolates from captive snakes

**Figure 1:**
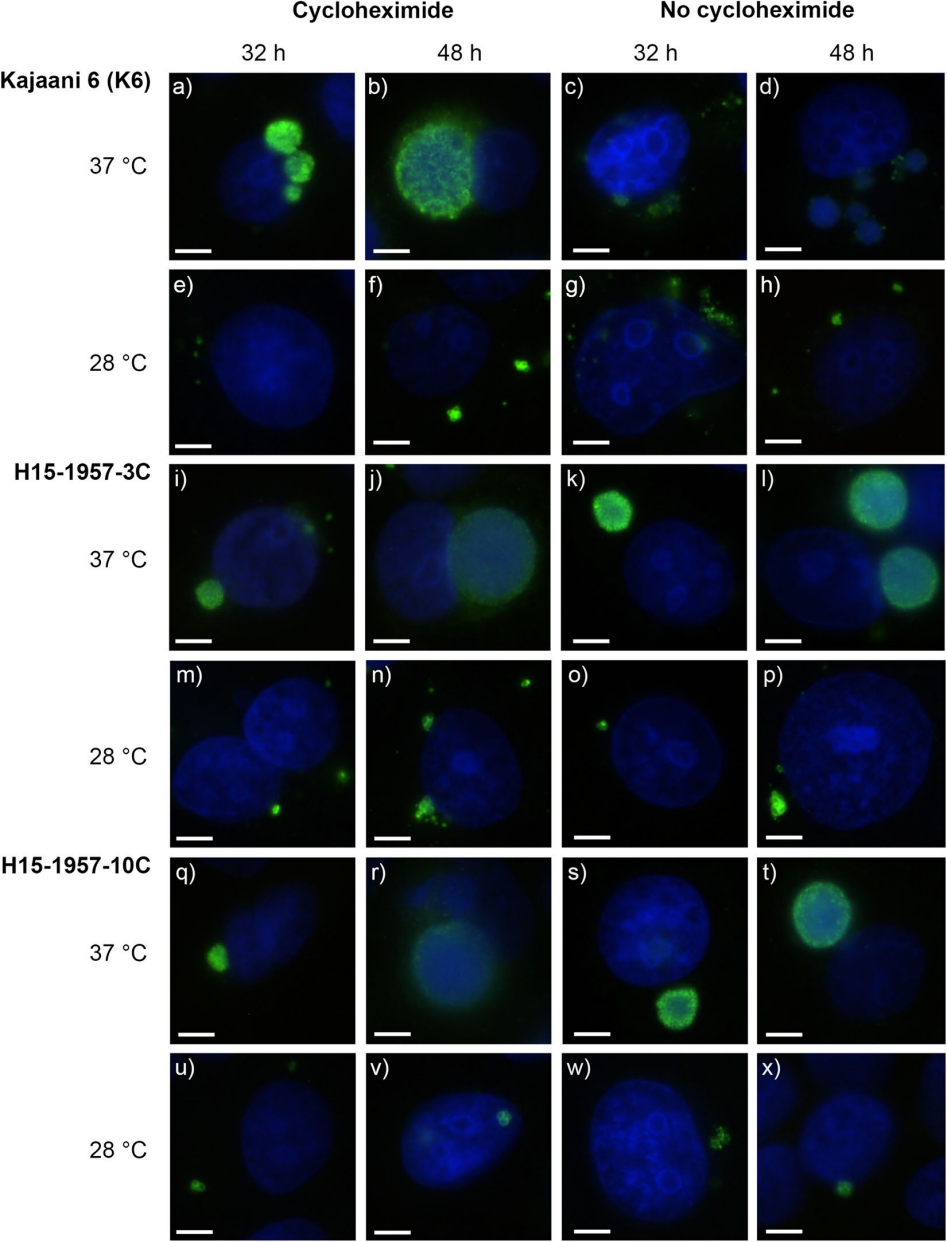
Immunofluorescence images of *C*. *pneumoniae* K6, H15-1957-3C and H15-1957-10C at 32 and 48 hours post infection (hpi). Shown are representative immunofluorescence images illustrating the morphology of *C*. *pneumoniae* K6 (**a**–**h**), H15-1957-3C (**i**–**p**), H15-1957-10C (**q**–**x**) at 32 hpi (columns 1 and 3) and 48 hpi (columns 2 and 4) in the presence (columns 1–2) or absence (columns 3–4) of cycloheximide. Strains were grown at 37 °C (top line per strain; lines 1, 3, 5) and 28 °C (bottom lane per strain; lines 2, 4, 6). The size bar indicates 5 µm. Chlamydial inclusions are shown in green, the LLC-MK2 nuclei (DAPI) are shown in blue.

**Figure 2:**
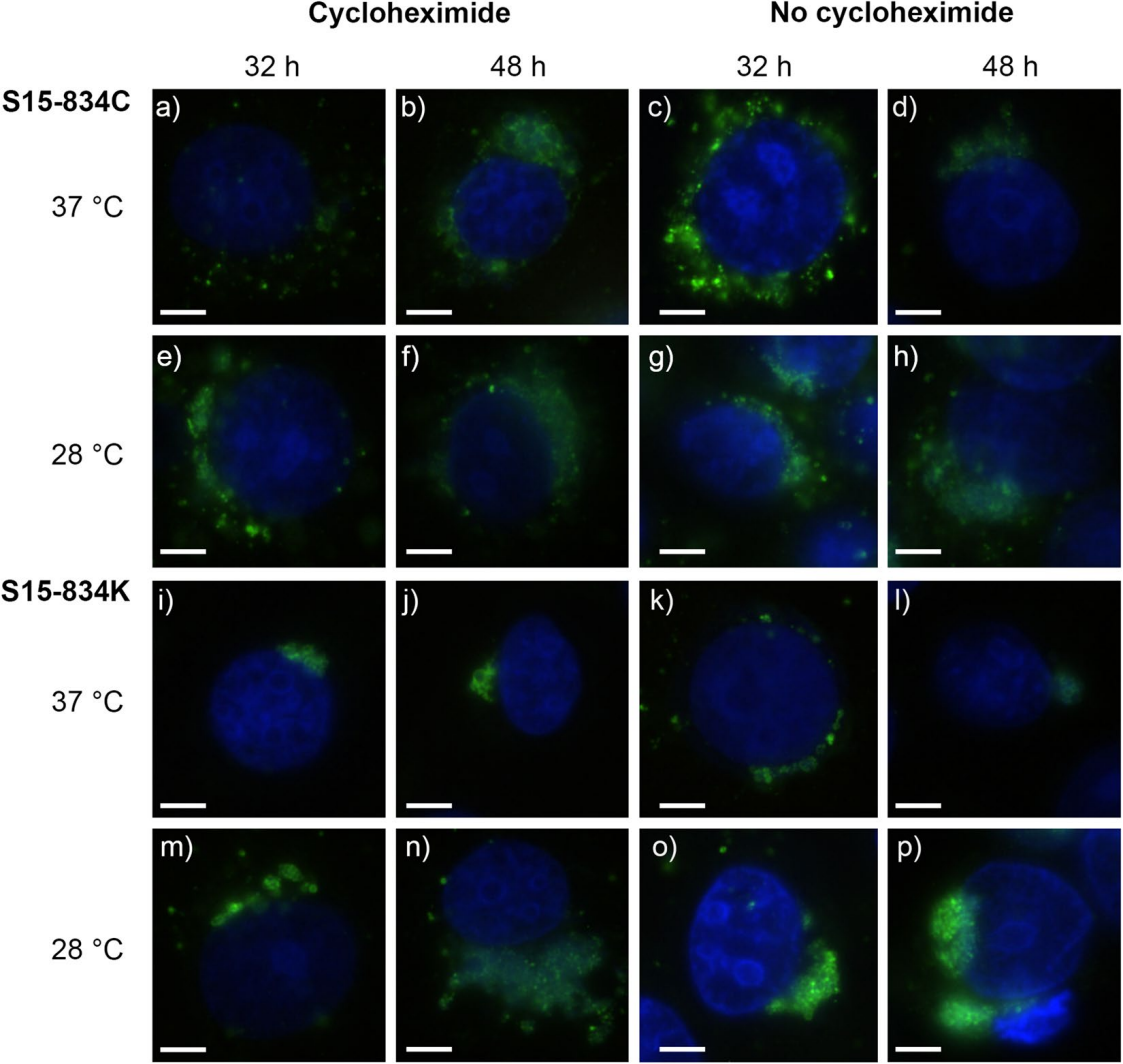
Immunofluorescence images of S15-834C and S15-834K at 32 and 48 hours post infection (hpi). Shown are immunofluorescence images illustrating the morphology of S15-834C (**a**–**h**) and S15-834K (**i**–**p**) at 32 hpi (columns 1 and 3) and 48 hpi (columns 2 and 4) in the presence (columns 1–2) or absence (columns 3–4) of cycloheximide. Strains were grown at 37 °C (top line per strain; lines 1, 3,) and 28 °C (bottom lane per strain; lines 2, 4,). The size bar indicates 5 µm. Chlamydial inclusions are shown in green, the LLC-MK2 nuclei (DAPI) are shown in blue.

Growth experiments were performed at 28 °C and 37 °C with and without cycloheximide for the four snake isolates, using the human *C*. *pneumoniae* strain K6 as a control. Initial growth curve experiments were performed using timepoints 24, 32, 40 and 48 hpi (Supplementary Figure S1), but revealed very small inclusions at 24 hpi rendering further evaluation difficult. Further experiments thus used only 32 and 48 hpi. Infected monolayers were evaluated for titre and by IF to determine inclusion morphology and size, at 32 and 48 hpi (from early reticular body (RB) phase to mature inclusions). Regardless of timepoint and temperature, the *C*. *pneumoniae* strain K6, isolates H15-1957-3C and H15-1957-10C formed larger inclusions at earlier timepoints when cycloheximide was added (Fig. 1) as compared to S15-834C and S15-834K (Fig. 2). At 37 °C, the *C*. *pneumoniae* strain K6 (Fig. 1, panel a–d), H15-1957-3C (Fig. 1, panel i–l) and H15-1957-10C (Fig. 1, panel q–t) formed round, regular inclusions over the course of 48 hours. At 28 °C, inclusions remained smaller at both investigated timepoints and contained mostly RB forms (Fig. 1, panels e–h, m–p, u–x).

In stark contrast, isolates S15-834C and S15-834K formed inclusions of heterogeneous appearance, mostly elongated and growing around the host cell nucleus in a crescent-like fashion (Fig. 2). At 37 °C, they regularly formed loosely connected, large granular RB-like structures (Fig. 2, panel a–d, i–l), whereas at 28 °C, granular inclusions consisting of EBs were seen at 48 hpi (Fig. 2, panels e–h, m–p).

**Figure 3:**
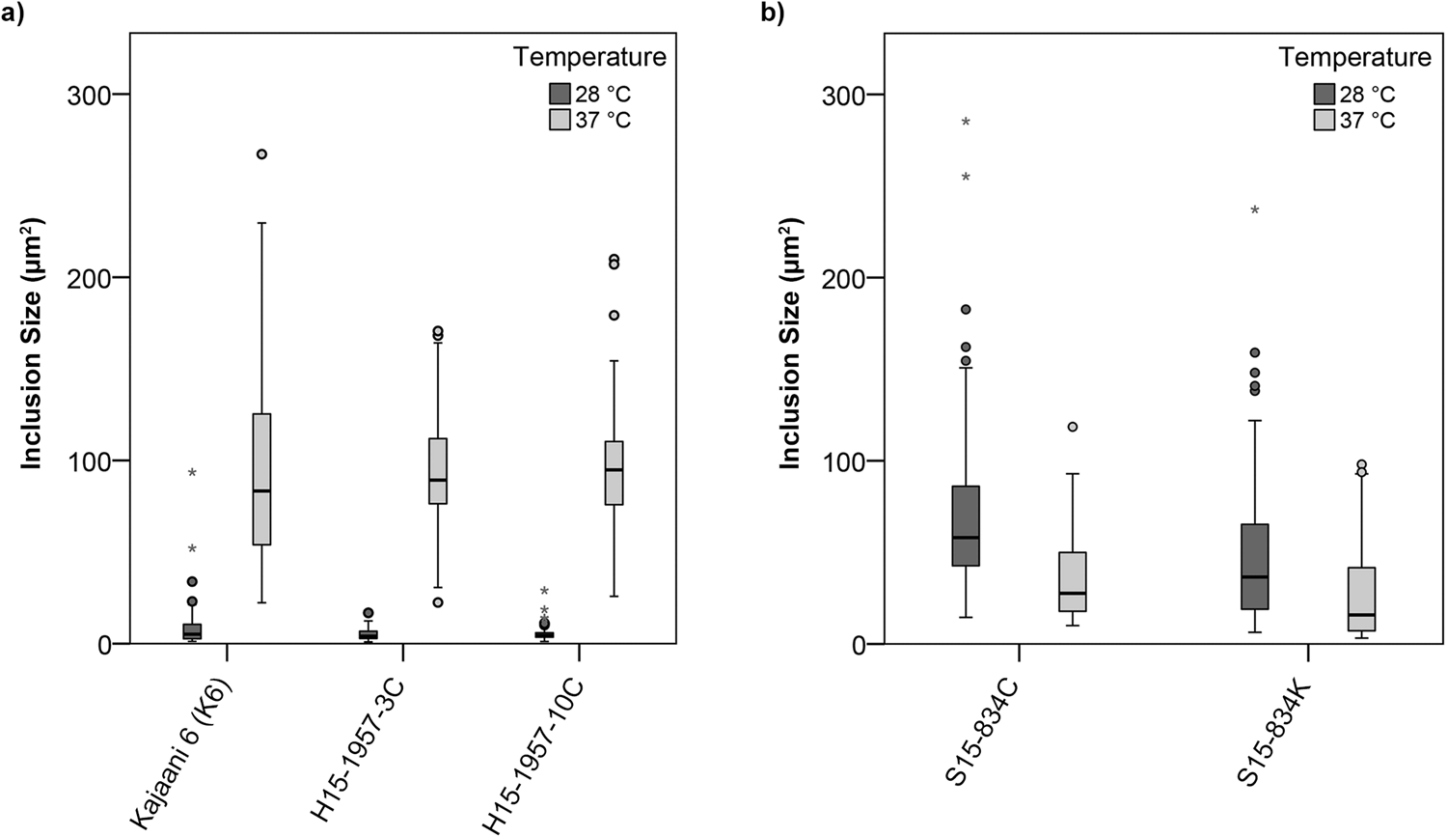
The average inclusion size of *C*. *pneumoniae* K6, H15-1957-3C and H15-1957-10C is significantly decreased following incubation at 28 °C compared to 37 °C. Shown is a boxplot comparing the inclusion size (µm^2^) distribution for strains a) *C*. *pneumoniae* K6, H15-1957-3C and H15-1957-10C, and b) S15-834C and S15-834K following 48 h of incubation at 28 °C (dark grey) or 37 °C (light grey). Filled circles represent outliers (>1.5x interquartile range), while asterisks represent extreme values (>3x interquartile range). The inclusion size was determined using a Leica DMLB fluorescence microscope (Leica Microsystems, Wetzlar, Germany) and a UI-2250SEC-HQ camera (uEye, IDS Imaging Development Systems GmbH, Obersulm, Germany) and analysed with the BonTec measuring and archiving software (BonTec, Bonn, Germany). Boxplots were created by the SPSS Statistics software. An unpaired *t* test was used for statistical analysis, and a p-value of less than 0.05 was considered significant.

Inclusion size was subsequently analysed at 48 hpi and compared between isolates and temperature (n = 50 inclusions per isolate and temperature analysed). Inclusion size for *C*. *pneumoniae* strain K6, H15-1957-3C and H15-1957-10C was significantly reduced at 28 °C when compared to 37 °C, with average sizes of 8.29 µm^2^ ± 1.48, 5.15 µm^2^ ± 0.55 and 5.15 µm^2^ ± 0.25 at 28 °C, and 94.19 µm^2^ ± 2.42, 94.09 µm^2^ ± 14.24 and 95.06 µm^2^ ± 17.11 at 37 °C (Fig. 3a). The average size of inclusions from isolates S15-834C and S15-834K was smaller at 37 °C than at 28 °C though the difference, while significant for S15-834K (p-value = 0.0278) was not significant for S15-834C (p-value = 0.0718): 34.19 µm^2^ ± 8.78 and 41.93 µm^2^ ± 3.28 at 37 °C and 71.79 µm^2^ ± 12.25 and 28.30 µm^2^ ± 0.00 at 28 °C (Fig. 3b).

**Figure 4:**
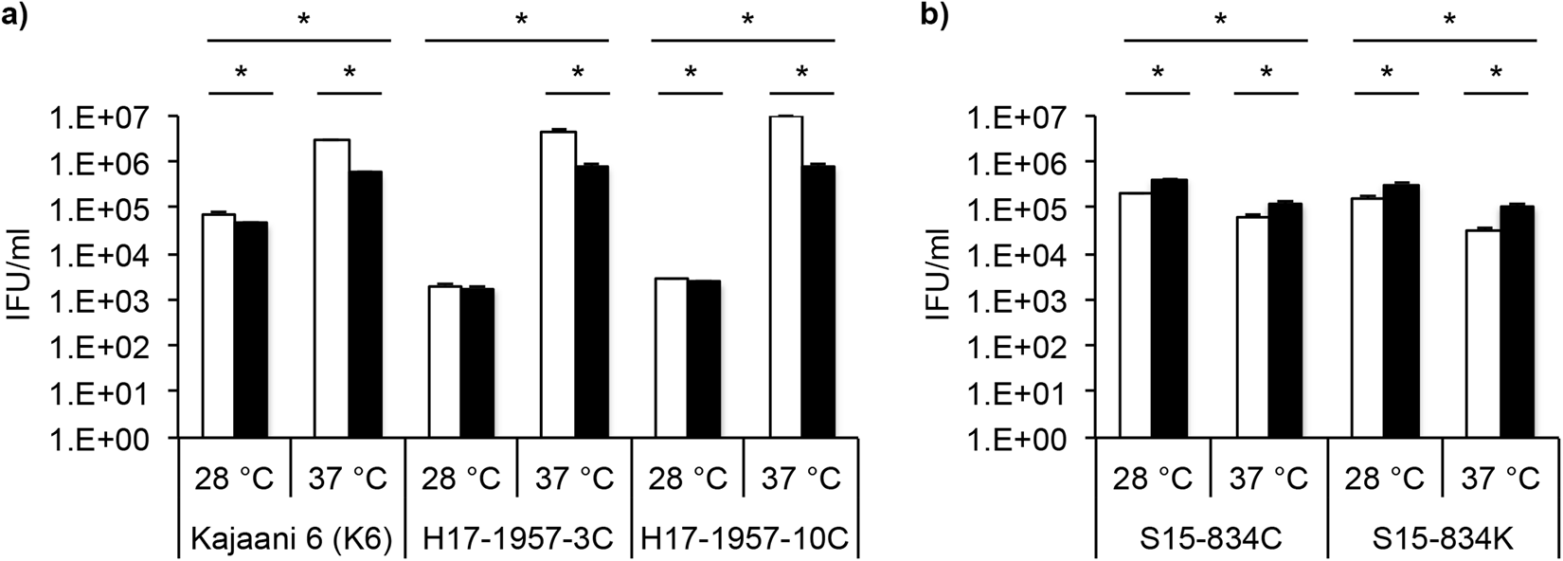
Titration by sub-passage confirmed that the infectivity of *C*. *pneumoniae* K6, H15-1957-3C and H15-1957-10C is significantly higher at 37 °C while the infectivity of strains S15-834C and S15-834K is increased at 28 °C. LLC-MK2 cells were infected with one chlamydial strain of a) either *C*. *pneumoniae* K6, H15-1957-3C, H15-1957-10C, or b) S15-834C, S15-834K and incubated for 48 hours at 28 °C or 37 °C in the presence (white bars) or absence (black bars) of cycloheximide. At 48 hours post infection, monolayers were scraped into the supernatant and used for titration by sub-passage. Inclusion forming units (IFU/ml) are shown in a logarithmic scale. Asterisks indicate statistical significance (p < 0.05) by unpaired *t* test.

Titration by sub-passage confirmed IF observations, showing that *C*. *pneumoniae* K6, H15-1957-3C and H15-1957-10C are significantly more infectious when grown at 37 °C compared to 28 °C. The addition of cycloheximide increased the infectivity in these strains (Fig. 4a). In contrast, the infectivity of S15-834C and S15-834K EBs was decreased after growth at 37 °C compared to 28 °C, and cycloheximide inhibited growth of these strains (Fig. 4b).

**Figure 5:**
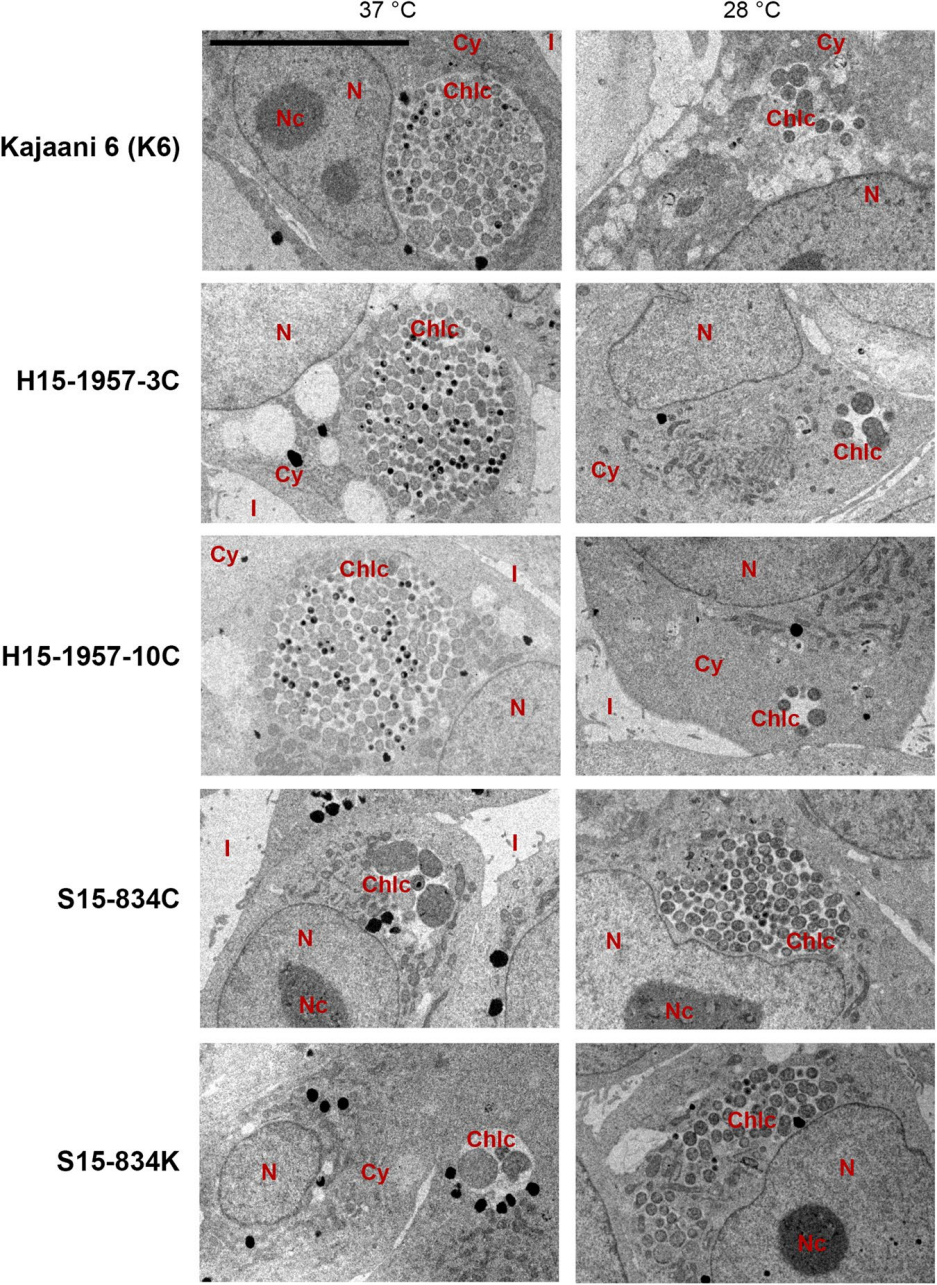
The ultrastructure of *C*. *pneumoniae* K6, H15-1957-3C and H15-1957-10C is similar with fully developed inclusions at 37 °C as opposed to 28 °C, while strains S15-834C and S15-834K form mature inclusions at 28 °C and show indication for the presence of aberrant bodies (ABs) at 37 °C. Cultures were fixed in glutaraldehyde at 48 hours post infection and processed for transmission electron microscopy. Shown is the ultrastructure of *C*. *pneumoniae* K6, H15-1957-3C, H15-1957-10C, S15-834C and S15-834K (top to bottom) following incubation at 37 °C (left column) or 28 °C (right column). The size bar indicates 10 µm. N = nucleus, Nc = nucleolus, Chlc = chlamydial inclusion, Cy = cytoplasm, I = intercellular space.

TEM of all isolates was performed at 48 hpi at 28 °C and 37 °C. The snake isolates displayed inclusions at various stages of the developmental cycle, similar in ultrastructure to *C*. *pneumoniae* strain K6 inclusions. In all four snake isolates, putative EBs are round, electron dense and, 0.25–0.5 nm in diameter. RBs are larger (0.5–1 mm), round to oval and more electron lucent. Binary fission of RBs and, occasionally, intermediate bodies (IBs, electron dense centre and more lucent periphery) were observed. Fully developed inclusions containing EBs, RBs and IBs were observed for the isolates at their respective optimum temperature, namely 37 °C for *C*. *pneumoniae* K6, H15-1957-3C and H15-1957-10C (Fig. 5, left panel) and 28 °C for S15-834C and S15-834K (Fig. 5, right panel). Interestingly, while inclusions at 28 °C resulted in small, RB-dominant inclusions for *C*. *pneumoniae* K6, H15-1957-3C and H15-1957-10C (Fig. 5, right panel), S15-834C and S15-834K also formed 2 µm and larger sized RB-like structures, which were interpreted as aberrant bodies (ABs; Fig. 5, left panel).

Antibiotic susceptibility to tetracycline, moxifloxacin and azithromycin was evaluated for the four snake isolates. The control *C*. *pneumoniae* strain K6 was sensitive to all tested antimicrobial substances with MIC/MBC values ranging from 0.06–0.5 μg/mL (Table 2). All snake isolates were equally sensitive to tetracycline and moxifloxacin but showed an intermediate to resistant phenotype to azithromycin (Table 2).

**Table 2:** Antibiotic susceptibility testing results of the four snake isolates and the human *C*. *pneumoniae* strain K6. Chlamydial isolates with a MIC/MBC of 4 μg/mL were defined as resistant, whereas cultures with 2 μg/mL ≤ MIC/MBC < 4 μg/mL were considered intermediate and isolates with a MIC/MBC of < 2 μg/mL were sensitive.

Isolate	Tetracycline (μg/mL)		Moxiflocaxin (μg/mL)		Azithromycin (μg/mL)	
	MIC	MBC	MIC	MBC	MIC	MBC
*C*. *pneumoniae* K6	0.125–0.25	0.25–0.5	0.06–0.125	0.06–0.125	0.25	0.25
H15-1957-3C	0.5–1	1–2	0.5–1	1	2	4
H15-1957-10C	0.25–0.5	0.5	<0.06	0.06	<4	<4
S15-834C	0.25	1	<0.06	0.06	2–4	>4
S15-823K	0.25	1	<0.06	0.06	2–4	>4

### Identification of the novel snake isolates

Initial identification using 16S rRNA genotype indicated that strains H15-1957-3C, H15-1957-10C are most closely related to *C*. *pneumoniae* (99% identity), whereas S15-834C and S15-834K are closer to *C*. *psittaci*, *C*. *caviae* and *C*. *abortus* (99% identity to each). Due to limitations with the resolution of 16S rRNA gene identification, and to gain further insights into the phylogenetic position and lifestyle of these bacteria, WGS was then performed on the strains used in the study, followed by finishing to generate complete chromosomes and manually improved automated annotation.

**Figure 6:**
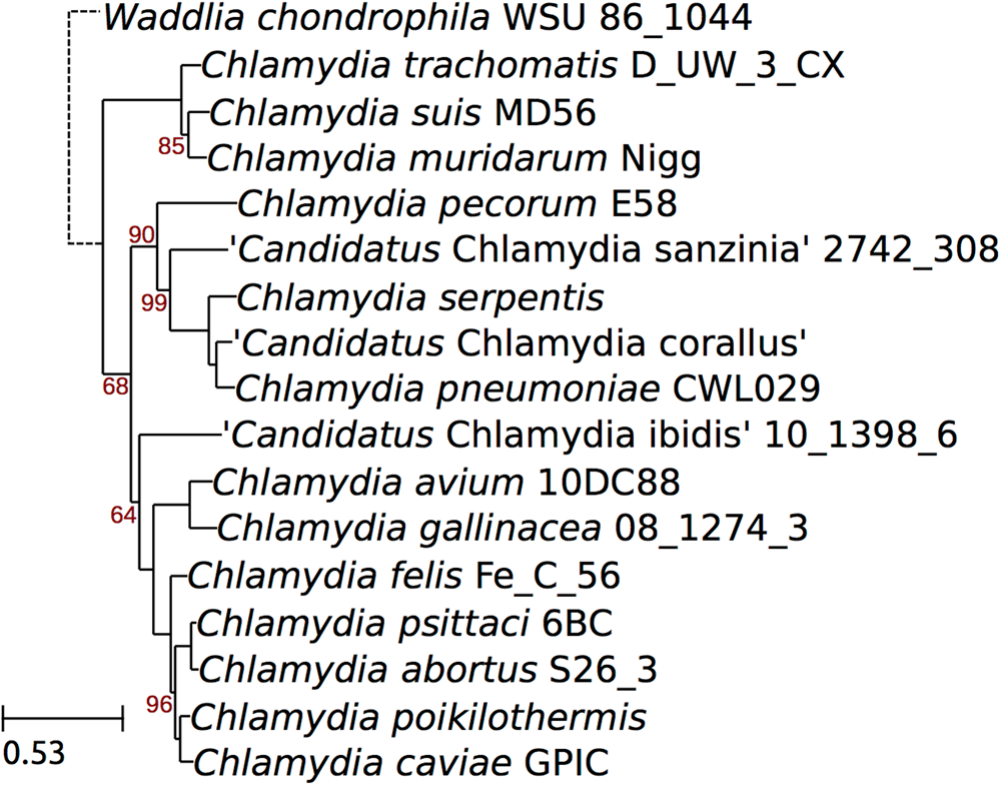
Phylogeny of the genus *Chlamydia* including the new species. The phylogeny was reconstructed based on the concatenated alignment of 9 phylogenetically informative protein markers^16^, and includes one representative of each species whenever genomic data is available. Bootstrap values are shown as percentages. The scale bar indicates the number of amino acid substitutions per site.

Using an established scheme based on nine conserved taxonomically informative gene products^16^, it was determined that the strains belong to two new species of the genus *Chlamydia*, with strains H15-1957-3C and H15-1957-10C most closely related to *C*. *pneumoniae*, and S15-834C and S15-834K most closely related to *C*. *caviae* (Fig. 6). Digital DNA-DNA hybridization (dDDH) analysis comparing their genomes to their respective closest relative species also indicated that these isolates represent novel species (22.6% and 30.9% respectively; Table 3). A scheme using pairwise comparisons of the nine gene products used above among all *Chlamydia* species, also confirmed these as new species^16^ (Supplementary Figure S2).

**Table 3:** Properties of genomes of novel species compared to these of close relatives and one other species isolated from a snake host. T3SS: type III secretion system, PLD: Phospholipase D.

Species	***C. pneumoniae***	***C. serpentis***	***C. caviae***	***C. poikilothermis***	***Ca. C. sanzinia***
**Host**	Human	Snake	Guinea pigs	Snake	Snake
**Disease**	pneumonia/bronchitis	undetermined	genital/ocular	undetermined	undetermined
**Strain**	CWL029	H15-1957-10C	GPIC	S15-834K	2742-308
**Accession number(s)**	AE001363	ERZ462351-2	AE015925-6	ERZ462353-4	CP014639
**Publication**	Kalman NG 1999 (41)	This publication	Read NAR 2003^39^	This publication	Taylor-Brown BMCG^14^
**Genome size**	1230230	1198609	1173390	1155104	1113073
**Predicted CDSs**	1073	985	1009	979	998
**Plasmid**	N (only in strain N16)	7533 bp	7966 bp	7559 bp	7504 bp
dDDH	22.60%		30.90%		
Novel species probability	98.50%		100%		
ANI genome	80.82%		86.01%		
**Virulence associated**					
ompA	Cpn_0695	C10C_0661	CCA_00047	C834K_0054	Cs308_0329/0497
T3SS	Y	Y	Y	Y	Y
tarp	Cpn_0572	C10C_0530	CCA_00170	C834K_0183	Cs308_0200
tarp repeats	N	Y	N	N	Y
pmp genes	22 (6 pseudogenes)	18	18 (1 pseudogene)	19 (3 pseudogenes)	19 (2 pseudogenes)
Locus_tags	Cpn_0005, 0013, 0444-7, 0451, 0453-4, 0466-7, 0471, 0539-40, 0963	C10C_0005,0007-8, 0410-4, 0416, 0419-20, 0424-7, 0495-6, 0936	CCA_00205-6, 00271-5, 00277-84, 00624, 00806	C834K_0221-2, 0291-7, 0299, 0302-5, 0642, 0828	Cs308_0065, 0068, 0078, 0080, 0163-4, 0586
pmp clusters	4	4	4	4	4
bioABD	Cpn_1041-4	C10C_1013-8	N - replaced by phage fragment	C834K_0742 (bioB)	Cs308_0663-6
udk/upp	Cpn_0735 (udk)	C10C_0705 (udk)	N	N	N
incA, B, C	Cpn_0186, 0585, 0291-2	C10C_0160, 0545, 0261-2	CCA_00550, 00491-0	C834K_0578, 0817, 0516-5	Cs308_0059, 0863-4
	Two incA homologues	Two incA homologues		Two incA homologues	
**PZ**/**RTR**					
accBC	Cpn_0182-3	C10C_0156-7	CCA_00553-4	C834K_0581-2	Cs308_0799-800
guaAB-add	Cpn_0170-2 (pseudogenes)	Cs0C_0149-52	CCA_00572-4	C834K_0590-2	N
cytotoxin	N	N	CCA_00558	N	N
PLD	N	N	N	N	N
MAC/perforin	Cpn_0176	C10C_0153	CCA_00560 (pseudogene)	N	N
trp operon	N	N	CCA_00562-7	N	N
Pseudogenes (curated)	Cpn_0014-5 (pmp)	C10C_0926 (ispE)	CCA_00285 (pmpG)	C834K_0289 (pmp)	Cs308_0073 (pmp)
	Cpn_0016-7 (pmp)		CCA_00288 (gatA)	C834K_0300 (pmp)	Cs308_0075 (pmp)
	Cpn_0018-9 (pmp)		CCA_00326 (hypothetical)	C834K_0310 (pmp)	
	Cpn_0170 (add)		CCA_00515 (hypothetical)	C834K_0638 (hypothetical)	
	Cpn_0171 (guaA)		CCA_00560 (MAC/perforin)	C834K_0657 (cons. hypothet)	
	Cpn_0267-8 (incA)		CCA_00886 (invasin)		
	Cpn_0339-41 (recF)				
	Cpn_0449-50 (pmp)				
	Cpn_0452 (pmp)				
	Cpn_0470 (pmp)				
	Cpn_0954-5 (ispE)				

These novel species were named *Chlamydia serpentis* (H15-1957-3C and H15-1957-10C), and *Chlamydia poikilothermis* (S15-834C and S15-834K). Careful comparisons of the genomes showed that the *C*. *serpentis* isolates H15-1957-3C and H15-1957-10C are identical, and the *C*. *poikilothermis* isolates S15-834C and S15-834K differ only by a single base pair indel (at 32260) within an intergenic homopolymeric tract.

### Whole genome characteristics of novel snake species

**Figure 7:**
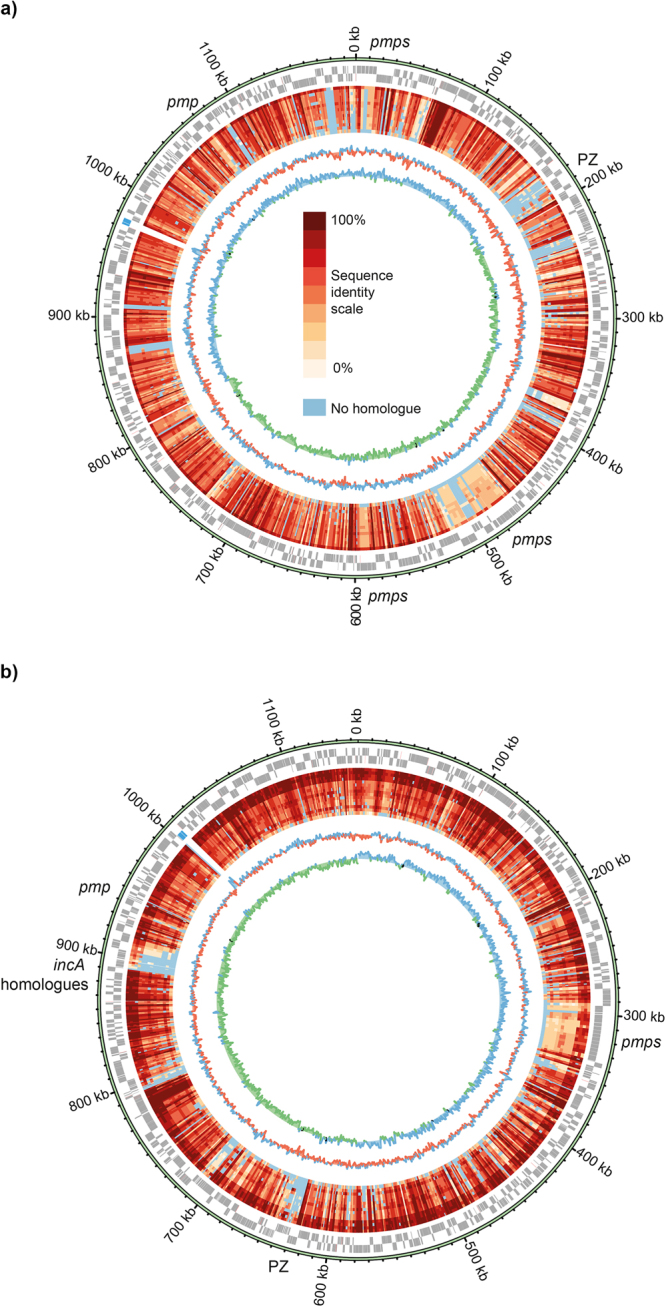
Circular representation of the chromosomes illustrating protein sequence conservation compared with other representatives of the *Chlamydiaceae*. The predicted CDSs are shown on the outer circle in forward or reverse frames. rRNA genes are shown in blue. The inner circles represent identities of the closest orthologue of each CDS identified using Orthofinder, compared against reference genomes. The genomes are ordered based on average sequence identity (see key) as below. Regions showing lower conservation are indicated. (**a**) *C*. *serpentis* H15-1957-10C: *C*. *pneumoniae* CWL029, *C*. *poikilothermis*. 834 K, *C*. *psittaci* 6BC, *C*. *felis* Fe/C-56, *C*. *caviae* GPIC, *Ca*. C. sanzinia 2742-308, *C*. *abortus* S26/3, *C*. *pecorum* E58, *C*. *gallinacea* 08–1274/3, *Ca*. C. ibidis 10–1398/6, *C*. *muridarum* Nigg, *C*. *trachomatis* D/UW-3/CX, *C*. *avium* 10DC88, *C*. *suis* MD56, *Waddlia chondrophila* WSU 86-1044. (**b**) *C*. *poikilothermis* S15-834K: *C*. *psittaci* 6BC, *C*. *caviae* GPIC, *C*. *felis* Fe/C-56, *C*. *abortus* S26/3, *C*. *serpentis* 10 C, *C*. *pneumoniae* CWL029, *Ca*. C. sanzinia 2742-308, *C*. *gallinacea* 08-1274/3, *Ca*. C. ibidis 10-1398/6, *C*. *pecorum* E58, *C*. *muridarum* Nigg, *C*. *trachomatis* D/UW-3/CX, *C*. *avium* 10DC88, *C*. *suis* MD56, *Waddlia chondrophila* WSU 86-1044. The two inner plots indicates the G + C content (blue for above average and red for below average) and G + C skew (blue for positive and green for negative).

Features of the genomes of these novel snake species are given in Table 3, compared against the most closely related species, and the other sequenced species from a snake host, *Ca*. C. sanzinia^14^. Both novel genomes were compared to representative genomes of fifteen species of the genus *Chlamydia* (Fig. 7a and b). Some regions of the chromosome exhibited lower sequence conservation as compared to other *Chlamydia* genomes, including the PZ, *pmp* operons and tandem arrays of repetitive genes (Fig. 7). Both the new species contain plasmids.

The genome of *C*. *serpentis* isolate H15-1957-10C is syntenic with that of the comparator *C*. *pneumoniae* CWL029, but has many whole gene differences, the overwhelming majority of which encode hypothetical proteins, including some families with *Chlamydia*-specific domains of unknown functions (DUF) (Supplementary Table S1). It also possesses fewer predicted pseudogenes than the genome of CWL02 (Table 3).

**Figure 8:**
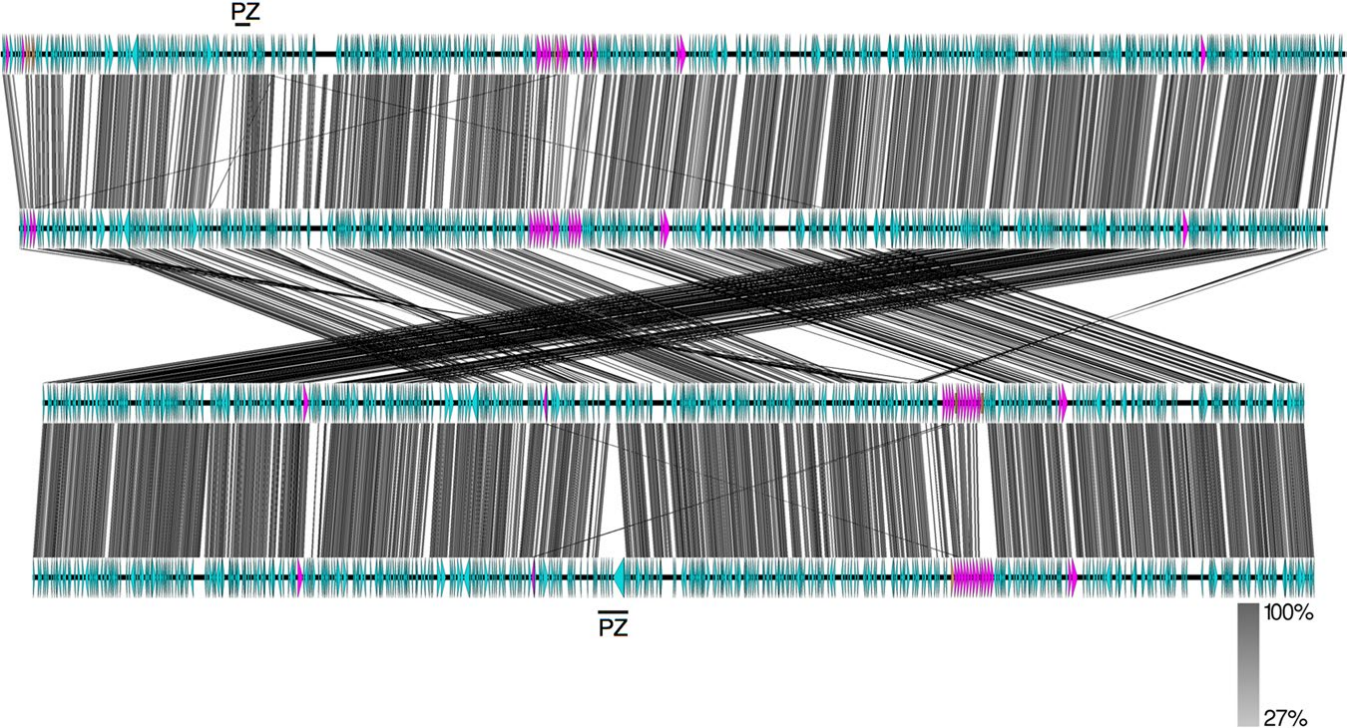
Comparison of the genomes of (top to bottom) *C*. *pneumoniae* CWL029, *C*. *serpentis* H15-1957-10C, *C*. *poikilothermis* S15-834K and *C*. *caviae* GPIC. Each horizontal line represents the genome, with CDSs shown directionally as arrow heads. Identity (tblastx) between the genomes is shown according to the scale bar. Pink CDSs represent *pmp* genes, and brown disrupted *pmp* genes. The genomes of S15-834K and *C*. *caviae* GPIC have been reverse complemented to illustrate the synteny. The plasticity zone (PZ) locus is indicated.

The genome of *C*. *poikilothermis* isolate S15-834K is syntenic with that of *C*. *caviae* GPIC (Fig. 8), showing very few whole gene differences. Most of the CDSs present in one strain and not the other encode hypothetical proteins (*C834K_0421*, *0529*, *0588*, *0725-7*, *0729*, *0816*, *and 0907-8; CCA_00082*, *00702*, *00707*) and some appear to reflect different numbers of tandemly arranged repetitive genes (*C834K_0358-60*, *0596-8*, *0652-6*, and *pmp* genes). In addition, the genome of *C*. *poikilothermis* isolate S15-834K possesses a CDS encoding a predicted alpha-rhamnosidase (*C834K_0688*), *bioB* (*C834K_00742*), replaced by phage gene fragments in *C*. *caviae* GPIC (*CCA_00721-2*; 17), and a second CDS encoding a putative IncA homologue (*C834K_0817*). The CDS representing a putative non-functional invasin (*CCA_00886*) is absent from the genome of *C*. *poikilothermis* isolate S15-834K.

The genomes of the novel species *C*. *serpentis* and *C*. *poikilothermis* each contain *pmp* genes in four clusters in the same genomic locations (Fig. 8). The genome of *C*. *pneumoniae* contains the highest number of *pmp* gene copies, but also the greatest number predicted to be inactivated.

Much of the variation between strains occurs within the plasticity zone (PZ), also known as the replication termination region (RTR). While both the novel snake species have *accBC* and *guaAB-add* gene clusters, often used to describe the ends of the PZ, neither carry genes encoding cytotoxins, phospholipase D, or the tryptophan operon. The *C*. *serpentis* genome of H15-1957-10C possesses a 2.5 kb putative MAC/perforin gene at *Csp10C2_00153*, and appears to have a PZ more intact than that found in the genomes of *C*. *pneumoniae* strains. In comparison, the *C*. *poikilothermis* genome of S15-834K has a reduced PZ relative to that within the genome of *C*. *caviae* GPIC.

To rationalize the azithromycin results for the snake strains, alignments of the 23S rRNA gene were performed, using sequences extracted from the genomes of S15-834K, H15-1957-10C, K6 and *C*. *caviae* GPIC. Of these, the former two possess intermediate resistance, and the latter two are sensitive. A single base substitution showing a distribution between these two groups was found, at base 1230 (AAGGAGTA/GCTGGAGC), which does not correlate with previous findings in which mutations at 2058 and 2059 have been implicated in conferring azithromycin resistance^17,18^.

## Discussion

### Evidence for the existence of two additional members of the genus Chlamydia

Analysis of four isolates originating from captive snakes of the species *Pantherophis guttatus* and *Atheris squamigera* has shown that the criteria for assignment of two new chlamydial species within the genus *Chlamydia* are fulfilled. First of all, 16S and 23S rRNA sequence identity to members of the order *Chlamydiales* was ≥80%, and sequence identity fulfilled the assignment to the family *Chlamydiaceae* (16S ≥ 92.5%, 23S ≥ 91%) according to the recent classification scheme by Pillonel *et al*.^16^. Based on the comparison of nine informative marker proteins with all other sequenced members of the *Chlamydia* genus, the new isolates can be assigned to two new species (RpoN < 96%, FtsK < 98%, PepF < 96%, Adk < 95%, HemL < 95%) of the the *Chlamydia* genus (DnA ≥ 70%, SucA ≥ 64%, Hyp325 ≥ 57% and Fabl ≥ 78%)^16^. Whole genome average nucleotide identity (ANI) analysis of the two new species with their closest relatives are also lower than the 95% generally considered to delineate bacterial species^19^ (Table 3). Previously, 16S and 23S rRNA sequences^20^ or multilocus sequence approaches^21^ were used to assign new chlamydial species. The scheme used in this study to classify novel snake isolates is based on a set of highly reliable protein sequences that were shown to be good markers of whole genome relatedness^16^.

Comparative genomic analysis shows that *C*. *serpentis*, most closely related to *C*. *pneumoniae*, appears to have a less disrupted genome than the latter, having a more extensive PZ and fewer pseudogenes, perhaps therefore being more representative of the common ancestor of the two species. *C*. *poikilothermis*, in contrast, has a more reduced PZ than the closest comparator *C*. *caviae*. Comparing with the genomes of other known *Chlamydia* found in snakes^14,15^, none carry the cytotoxin or *trp* operon in the PZ, possibly implying that this improves their colonisation of snakes. Presence of the purine biosynthesis genes *guaAB-add*, however, is not required for growth within snakes, as this operon is absent from *Ca*. C. sanzinia.

Both novel species behaved intermediate to resistant to azithromycin. According to Ellington *et al*.^22^ and Tagini *et al*.^23^, the genotypic to phenotypic congruence for antibiotic susceptibility may vary quite extensively from species to species and from antibiotics to antibiotics. Thus, the fact that these new chlamydia-related species exhibits a low susceptibility to azithromycin in absence of the typical mutations described in other species is somehow expected and highlights the limitation and difficulties faced when trying to infer AB susceptibility from genomic data.

### Description of *C*. *serpentis* sp. nov. (H15-1957-10C)

*C*. *serpentis* (serpentis L. fem. Gen. pl. *serpentum*, of the snake, because snakes are the currently known host).

*C*. *serpentis* strains occur in snakes belonging to the families *Colubridae* and *Viperidae* and were isolated from captive asymptomatic *Patherophis guttatus* and *Atheris squamigera*. The presence of the agent in other snake species as well as in free-ranging snakes and even other reptiles seems possible, but has yet to be investigated. *C*. *serpentis* can be recovered from choanal and cloacal swabs and might be also detected in inner organs of infected reptiles. The natural route of transmission and potential reservoirs are unknown to date. The carrier snakes were clinically asymptomatic, but a facultative pathogenic role has to be considered in concert with other bacterial or viral infections, or induced by stress due to capture and transportation, high-density farming and hibernation^12^. The potential for zoonotic infection of humans, in particular snake owners, is unknown.

*C*. *serpentis* can be grown in LLC-MK2 cells, a rhesus monkey epithelial kidney cell line, which has been successfully used to isolate *C*. *suis* strains from fecal swab samples^24^ and is able to survive and replicate at lower temperature such as 28 °C and 12 °C. The replication of *C*. *serpentis* is enhanced by adding cycloheximide after the infection to block *de novo* host protein synthesis similar as shown for *C*. *pneumoniae* K6. Shape, size and distribution of inclusions including their production of infectious EBs measured as IFU per mL at 32 and 48 hpi resemble those seen in *C*. *pneumoniae*-infected LLC-MK2 cells. Size of inclusions and productivity of *C*. *serpentis* at 28 °C is diminished compared to 37 °C in line with *C*. *pneumoniae* K6 in this study. By TEM, the typical bi-phasic developmental cycle can be observed for *C*. *serpentis* including EBs and RBs comparable in size and morphology to *C*. *pneumoniae*. *C*. *serpentis* is susceptible to tetracycline and moxifloxacin but has an intermediate sensitivity of azithromycin (ranging from 2 to more than 4 μg/mL). The type strain is H15-1957-10C^T^. Two strains H15-1957-10C (DSM 106151) and H15-1957-3C (DSM 106152) have been deposited at the DSMZ (Deutsche Sammlung von Mikrorganismen und Zellkulturen GmbH, Braunschweig, Germany) and at CSUR (Collection de Souches de l′ Unité Rickettsies WDCM 875, Marseille, France).

### Description of C. poikilothermis sp. nov. (S15-834K)

*C*. *poikilothermis* (poikilothermis adj., of a poikilotherm species, because this species was isolated from a poikilotherm reptile which is an organism whose internal temperature varies considerably).

Two highly similar strains of this species occurred in a captive *Pantheropis guttatus* belonging to the family *Colubridae*. The agent can be recovered from choanal and cloacal swab and is possibly also present in other snake families, captive or free-ranging. A pathogenic potential cannot be differentiated from the cause of death (salmonellosis) in the actual case. The mode of transmission and zoonotic potential are unknown. Like other *Chlamydiaceae* species, *C*. *poikilothermis* can be isolated and grown in cell culture but requires lower temperatures such as 28 °C. Isolation at 37 °C is less successful, growth curves (Supplementary Figure S1) over time show the ability of *C*. *poikilothermis* to replicate at 37 °C but inclusions are significantly smaller and morphologically similar to ABs. The replication of *C*. *poikilothermis* is not enhanced by adding cycloheximide. It grows better in the absence of cycloheximide regardless of the temperature (28 °C, 37 °C). By IF, the inclusion morphology at 28 °C is heterogenous and inclusions tend to grow around host cell nuclei. The ultrastructural features of *C*. *poikilothermis* display EBs and RBs replicating by binary fission.

*C*. *poikilothermis* is susceptible to tetracycline and moxifloxacin but behaves intermediate to resistant to azithromycin (ranging from 2–4 μg/mL). The type strain is S15-834K^T^. Two strains S15-834K (DSM 106149) and S15-834C (DSM 106150) have been deposited at the DSMZ (Deutsche Sammlung von Mikrorganismen und Zellkulturen GmbH, Braunschweig, Germany) and at CSUR (Collection de Souches de l′ Unité Rickettsies WDCM 875, Marseille, France).

## Material and Methods

### Snake collections, sample collection and DNA extraction

Samples (n = 23) investigated in this study comprised choanal and cloacal swabs (FLOQSwabs®, Copan Italia, Brescia, Italy) taken from 18 captive snakes belonging to the families *Boidae*, *Colubridae*, *Pythonidae* and *Viperidae*. The majority of the snakes was sampled in a previous study^11^ and included six private snake collections in Switzerland. These snakes were clinically inconspicuous at the time of sampling. Samples were collected as dry swabs for DNA extraction and subsequent chlamydial screening. *Chlamydia*-positive snakes were sampled again, whereas swabs were stored in sucrose phosphate (SP) transport medium at −80 °C for isolation as described^24^. Additionally, two individual captive snakes, which had been submitted to the Institute of Veterinary Pathology (Vetsuisse Faculty, University of Zurich) for diagnostic purposes, were sampled during necropsy. Two swabs were collected per snake and anatomical location, of which one swab was stored in SP medium.

### Chlamydial screening

DNA of dry swab samples was extracted using the QIAamp DNA mini kit (Qiagen, Hilde, Germany), following the supplier’s recommendations. Extracted DNA of all samples (n = 23) was examined using real-time PCR based on *Chlamydiaceae* family-specific 23S rRNA gene primers performed on an ABI 7500 instrument, as previously described^25^ including internal amplification controls^26^. All samples were tested in duplicate and the cycle threshold was set at 0.1 for each run. A mean cycle threshold (Ct value) < 38 was considered positive, and was used to calculate the corresponding chlamydial load as *Chlamydiaceae* 23S rRNA gene copy number per µl. If the amplification of internal control DNA was inhibited, the run was repeated following 1:10 dilution of the sample. A positive control containing a sevenfold dilution series of *C*. *abortus* DNA and a negative control of water instead of the template DNA were included in each run^27^.

All samples (n = 23) were further investigated using a species-specific 23S rRNA Arraymate microarray assay (Alere, Jena, Germany), as established by Borel *et al*.^28^. The current version carries 34 probes for eleven *Chlamydiaceae* species, three genus-specific probes, four family markers and 15 probes for *Chlamydia*–like organisms. Additionally, there are four internal control DNA probes and an internal staining control (biotin marker)^27^. Each sample, including internal control DNA (Intype IC-DNA, Qiagen Labor, Leipzig, Germany), was amplified and biotin-labeled using a biotinylation PCR, as described by Borel *et al*.^28^, with 10 minutes (min) of initialization (96 °C) and 40 cycles of 94 °C (denaturation), 50 °C (annealing), and 72 °C (elongation) for 30 seconds each. 2–4 µl of amplification product was loaded on the chip, which was processed according to manufacturer’s instructions.

### Isolation of Chlamydia

Isolation from swabs in SP medium was attempted on 18 swabs from 13 snakes positive by chlamydial screening (Table 1) as described in Wanninger *et al*.^24^ with minor changes. Briefly, the isolation process was performed in LLC-MK2 cells (continuous Rhesus monkey kidney cell line, provided by IZSLER Brescia, Italy) grown on glass coverslips (∅ 12 mm) in 7 ml Trac bottles (Thermo Fisher Scientific, Waltham, MA, USA) at 37 °C and 5% CO_2._ Swab samples were thawed in SP medium, vortexed with glass beads for 1 min and used to inoculate LLC-MK2 cells. Infected cells were then centrifuged for 3 hours (h) at 2385 g (28 °C or 33 °C) before incubation for 48 to 72 h at 37 °C. If enough SP material was available, incubation was additionally performed at 28 °C in a second isolation attempt. Cultures were considered negative for viable *Chlamydia* if no inclusions were detected after three passages.

### Inclusion morphology and growth characteristics of novel snake chlamydiae

Isolated snake chlamydial strains H15-1957-3C, H15-1957-10C, S15-834C and S15-834K were further characterized *in vitro* using LLC-MK2 cells, including *C*. *pneumoniae* Kajaani 6 (K6) as a control (kindly provided by Dr Claudia Dumrese and Dr Urs Ziegler, Center for Microscopy and Image analysis, University of Zurich, Switzerland). LLC-MK2 cells, seeded at a density of 2.5 × 10^5^ cells per well and cultivated overnight in 24-well plates in growth medium (500 ml Eagle’s minimum essential medium [EMEM, Gibco, Thermo Fisher Scientific, Invitrogen, Carlsbad, CA, USA] supplemented with 10% heat-inactivated fetal calf serum [FCS, BioConcept, Allschwil, Switzerland], 5 ml L-glutamine (100x Glutamax, Gibco) and 6 ml D-(+)-glucose [0.06 g/ml, Sigma-Aldrich Co., St. Louis, MO, USA]), were infected with either of the five strains at multiplicity of infection (MOI) of either 0.5 (H15-1957-3C and H15-1957-10C) or 1 (*C*. *pneumoniae* K6), or using an inoculum (S15-834C and S15-834 K) to reach an infectivity of approximately 75% after 48 hours post infection (hpi). Incubation medium, used for inoculating and incubation of cells with *Chlamydia*, consisted of EMEM supplemented with 20% FCS, 2 g glucose, 5 ml L-glutamine, 4 ml Fungizone (250 µg/ml, Gibco)^24^. Infected monolayers were centrifuged for 1 h at 1000 g and 25 °C. After centrifugation, inocula were replaced by fresh incubation medium with or without 1.4 μg/ml cycloheximide (Sigma-Aldrich, St. Louis, MO, USA). Duplicate infected cultures were incubated at 28 °C and 37 °C and 5% CO_2_ for 24, 32, 40 and 48 hours for preliminary evaluations. At indicated timepoints, infected cells were further processed for indirect immunofluorescence microscopy (IF), titre analysis and transmission electron microscopy (TEM).

For IF, cells were fixed with absolute methanol (−20 °C) for 10 min and chlamydial inclusions were visualized using a *Chlamydiaceae* family-specific mouse monoclonal antibody directed against the chlamydial lipopolysaccharide (LPS, Clone ACI-P, 1:200; Progen, Heidelberg, Germany) and 1:500 diluted Alexa Fluor 488-conjugated secondary goat anti-mouse antibody (Molecular Probes, Eugene, OR, USA). Host and chlamydial DNA were labelled using 1 μg/ml 4′,6-diamidino-2′-phenylindole dihydrochloride (DAPI, Molecular Probes). Coverslips were mounted with FluoreGuard mounting medium (Hard Set; ScyTek Laboratories Inc., Logan, UT, USA) on glass slides and evaluated using a Leica DMLB fluorescence microscope (Leica Microsystems, Wetzlar, Germany) under oil immersion at 1000x magnification with a 10x objective (PL FLUOTAR 100x/1.30, OIL, ‘/0.17/D, Leica Microsystems) and a 10x ocular objective (Leica L-Plan 10x/25 M, Leica Microsystems) as described^29^. To determine the mean inclusion size, images were taken using a Leica DMLB fluorescence microscope (Leica Microsystems, Wetzlar, Germany) and a UI-2250SEC-HQ camera (uEye, IDS Imaging Development Systems GmbH, Obersulm, Germany) and analysed with the BonTec measuring and archiving software (BonTec, Bonn, Germany). Depending on the inclusion size, a magnification of 1000x (oil immersion) or 40x was chosen and the area was measured with either the “Fläche(Kreis) [Area(circle)]” or “Fläche(Polygon) [Area(polygon)]” function depending on the shape of the inclusion. The area of at least 50 inclusions was measured from one or two coverslips in one or two independent experiments. Boxplots were created with the SPSS Statistics software. The QuickCalcs unpaired *t* test of the GraphPad software (https://www.graphpad.com/quickcalcs/ttest1.cfm) was used for statistical analysis.

For titre analysis, infected monolayers were scraped into 1 ml fresh infection medium at 32 and 48 hpi. Supernatant and scraped cells were pooled before storage at −80 °C. Chlamydial titration by sub-passage was evaluated to determine the infectivity of the isolated strains at their respective optimum temperature (*C*. *pneumoniae* K6, H15-1957-3C and H15-1957-10C at 37 °C; S15-834C and S15-834K at 28 °C) after 48 hpi. As previously described^24^, samples were vortexed for 1 min before serial dilution in incubation medium and subsequent infection of the prepared LLC-MK2 cells was carried out as described for infection of host cells. Fixation and immunostaining was performed as described for IF. The number of inclusions in 30 random microscopic fields for duplicate coverslips per condition was determined using a Leica fluorescence micro- scope at 200x magnification with a 20x objective (PL FLUOTAR 20x/0.50 PH 2, ‘/0.17/B) and a 10x ocular objective (Leica L-Plan 10x/25 M, Leica Microsystems). Inclusion forming units (IFU) per ml of undiluted inoculum was then calculated.

For TEM analysis, cells were fixed with 2.5% glutaraldehyde (Electron Microscopy Sciences, Ft. Washington, USA) for 1 h and embedded in epoxy resin (Fluka; Sigma-Aldrich).

### Antibiotic susceptibility testing

Antibiotic susceptibility testing of the four snake isolates (H15-1957-3C, H15-1957-10C, S15-834C and S15-834K) and the human *C*. *pneumoniae* isolate K6 was performed as described previously^24,30^. Antimicrobial drugs included tetracycline (Sigma-Aldrich), moxifloxacin (Sigma-Aldrich) and azithromycin (Sigma-Aldrich).

Briefly, we collected chlamydiae-positive cultures in SP media and determined the number of inclusion forming units per ml (IFU/ml) via 10-fold dilution as described^24,30^ Each antibiotic susceptibility determination was performed with approximately 5 × 10^3^ IFU/mL per investigated isolate^30,31^. Following inoculation of sixteen confluent monolayers and centrifugation (1 h, 2385 g, 33 °C), inocula were replaced with incubation medium containing a serial two-fold dilution of the antimicrobial agent in question. After a 48-hour incubation period at their optimum temperature (28 °C or 37 °C), monolayers were fixed in methanol for 10 min, immunolabelled and processed with a fluorescein-conjugated monoclonal antibody specific for the chlamydial LPS genus-specific antigen (IMAGEN Chlamydia K610111–2, Thermo Fisher Scientific) as described^24^.

The minimum inhibitory concentration (MIC) of the respective antibiotic substance was defined as “the lowest concentration preventing the detection of more than 90% of the chlamydial inclusions compared with the drug-free control”^30,31^. In parallel, media of the remaining Trac bottles was replaced with antibiotic-free chlamydiae cultivation medium after washing the coverslips with PBS. Monolayers were fixed after 48 h of incubation and immunolabelled for the evaluation of the minimum bactericidal concentration (MBC), which was identical to MIC determination. Chlamydial isolates with an MIC/MBC of 4 μg/mL were defined as resistant, whereas cultures with 2 μg/mL ≤ MIC/MBC < 4 μg/mL were considered intermediate, and isolates with a MIC/MBC of <2 μg/mL sensitive.

### Identification using 16S rRNA gene analysis

Initial genotyping was performed by sequencing the 16S rRNA gene using the *Chlamydiales*-specific primer pair 16SIGF (5′- CGGCGTGGATGAGGCAT-3′) and 16SB1 (5′-TACGGYTACCTTGTTACGACTT-3′)^32^ targeting almost the entire gene (approximately 1400 bp) as described^11^. PCR products were purified with the QIAquick PCR Purification Kit (Qiagen) according to manufacturer’s instructions. Purified DNA was Sanger sequenced by Microsynth (Balgach, Switzerland).

### WGS, mapping, assembly and annotation

Sequencing of the four snake isolates (H15-1957-3C, H15-1957-10C, S15-834C and S15-834K) and the human *C*. *pneumoniae* isolate K6 was performed on the Illumina Miseq platform with 250 bp paired end reads at the Functional Genomics Center Zurich (FGCZ), following NEBNext library creation. Coverage data is shown in Supplementary Table S2. Assembly was performed using SPAdes in multi-cell mode^33^, followed by ordering of the chlamydial contigs against *C*. *pneumoniae* CWL029 (accession number AE001363) or *C*. *psittaci* 01DC12 (HF545614) within ACT^34^. The contig order was confirmed, and genomes finished, by amplifying and capillary sequencing across gaps using the primers listed in Supplementary Table S3. The resulting single contig genome assemblies were checked and compared using BWA^35^. The genome of K6 remained in two contigs. Automated annotation was performed using AnnotateBacteria [https://github.com/sanger-pathogens/Bio-AutomatedAnnotation/] with further manual curation in Artemis^34,36^. All read data, with associated assembly and annotation where relevant, has been submitted to ENA under project PRJEB19768.

### Phylogenetic and genome analysis

Average nucleotide identity (ANI) determination was performed at enve-omics.ce.gatech.edu/ani/^19^ and digital DNA-DNA hybridisation (dDDH) using GGDC2.1 (ggdc.dsmz.de/distalcalc2.php) and the DDH cut off of ≤70%^37^. Comparator genomes used were *C*. *pneumoniae* CWL029 (accession number AE001363)^38^ and *C*. *caviae* GPIC (AE015925)^39^. Comparisons were performed using tblastx, visualized in ACT, to identify novel and duplicated genes.

Nine phylogenetically informative markers were identified with hmmsearch v3.1 (http://journals.plos.org/ploscompbiol/article?id=10.1371/journal.pcbi.1002195) using hmm profiles built from 21 reference sequences^16^. Amino acid sequences were aligned with mafft (parameters:–auto–maxiterate 1000). Alignments were concatenated to build a reference phylogeny using Phyml v 3.1 (https://www.ncbi.nlm.nih.gov/pubmed/20525638), with the LG + Г + I model and 100 bootstrap replicates. Pairwise amino acid sequence identities were calculated based on pairwise Needleman-Wunsch global alignments (EMBOSS:6.6, https://www.ncbi.nlm.nih.gov/pubmed/10827456). Amino acid sequences of predicted coding sequences were clustered into orthologous groups with Orthofinder v. 0.4 (https://genomebiology.biomedcentral.com/articles/10.1186/s13059–015–0721–2).

## Electronic supplementary material

Supplementary files
