# Author Correction: Novel *Chlamydia* species isolated from snakes are temperature-sensitive and exhibit decreased susceptibility to azithromycin

**DOI:** 10.1038/s41598-021-97589-6

**Published:** 2021-09-08

**Authors:** Eveline Staub, Hanna Marti, Roberta Biondi, Aurora Levi, Manuela Donati, Cory Ann Leonard, Serej D. Ley, Trestan Pillonel, Gilbert Greub, Helena M. B. Seth-Smith, Nicole Borel

**Affiliations:** 1 Institute for Veterinary Pathology, Vetsuisse Faculty, University of Zurich, Winterthurerstrasse 268, CH-8057 Zurich, Switzerland; 2 DIMES, Microbiology, Policlinico S. Orsola, University of Bologna, 40138 Bologna, Italy; 3 Institute of Microbiology, University of Lausanne, CH-1011 Lausanne, Switzerland; 4 Applied Microbiology Research, Department of Biomedicine, University of Basel, Basel, Switzerland; 5 Present Address: SA MRC Centre for Tuberculosis Research, DST/NRF Centre of Excellence for Biomedical Tuberculosis Research, Division of Molecular Biology and Human Genetics, Stellenbosch University, Stellenbosch, South Africa; 6 Present Address: Clinical Microbiology, University Hospital Basel, Basel, Switzerland

Correction to: *Scientific Reports* 10.1038/s41598-018-23897-z, published online 04 April 2018

The original version of this Article contains errors.

The description of *Chlamydia serpentis* sp.nov and *Chlamydia poikilotherma* sp. nov was incomplete.

Those species are validated herein:

**Description of*Chlamydia. serpentis*** **sp. nov.**

(ser.pen’tis. L. gen. n. *serpentum*, of the snake)

*C*. *serpentis* strains occur in snakes belonging to the families *Colubridae* and *Viperidae* and were isolated from captive asymptomatic *Patherophis guttatus* and *Atheris squamigera*. The presence of the agent in other snake species as well as in free-ranging snakes and even other reptiles seems possible, but has yet to be investigated. *C*. *serpentis* can be recovered from choanal and cloacal swabs and might be also detected in inner organs of infected reptiles. The natural route of transmission and potential reservoirs are unknown to date. The carrier snakes were clinically asymptomatic, but a facultative pathogenic role has to be considered in concert with other bacterial or viral infections, or induced by stress due to capture and transportation, high-density farming and hibernation^12^. The potential for zoonotic infection of humans, in particular snake owners, is unknown.

*C*. *serpentis* can be grown in LLC-MK2 cells, a rhesus monkey epithelial kidney cell line, which has been successfully used to isolate *C*. *suis* strains from fecal swab samples^24^ and is able to survive and replicate at lower temperature such as 28 °C and 12 °C. The replication of *C*. *serpentis* is enhanced by adding cycloheximide after the infection to block *de novo* host protein synthesis similar as shown for *C*. *pneumoniae* K6. Shape, size and distribution of inclusions including their production of infectious EBs measured as IFU per mL at 32 and 48 hpi resemble those seen in *C*. *pneumoniae*-infected LLC-MK2 cells. Size of inclusions and productivity of *C*. *serpentis* at 28 °C is diminished compared to 37 °C in line with *C*. *pneumoniae* K6 in this study. By TEM, the typical bi-phasic developmental cycle can be observed for *C*. *serpentis* including EBs and RBs comparable in size and morphology to *C*. *pneumoniae*. *C*. *serpentis* is susceptible to tetracycline and moxifloxacin but has an intermediate sensitivity of azithromycin (ranging from 2 to more than 4 μg/mL).

The type strain H15-1957-10C^T^ (DSM 106151, CSUR Q5983) and reference strain H15-1957-3C (DSM 106152, CSUR Q5983) have been deposited at the DSMZ (Deutsche Sammlung von Mikrorganismen und Zellkulturen GmbH, Braunschweig, Germany) and at CSUR (Collection de Souches de l′ Unité Rickettsies WDCM 875, Marseille, France).

**Description of*Chlamydia. poikilotherma*** **sp. nov.**

(poi.ki.lo.ther’ma. N.L. fem. adj. *poikilotherma* poikilothermic)

Two highly similar strains of this species occurred in a captive *Pantheropis guttatus* belonging to the family *Colubridae*. The agent can be recovered from choanal and cloacal swab and is possibly also present in other snake families, captive or free-ranging. A pathogenic potential cannot be differentiated from the cause of death (salmonellosis) in the actual case. The mode of transmission and zoonotic potential are unknown. Like other *Chlamydiaceae* species, *C*. *poikilotherma* can be isolated and grown in cell culture but requires lower temperatures such as 28 °C. Isolation at 37 °C is less successful, growth curves (Supplementary Figure S1) over time show the ability of *C*. *poikilotherma* to replicate at 37 °C but inclusions are significantly smaller and morphologically similar to ABs. The replication of *C*. *poikilotherma* is not enhanced by adding cycloheximide. It grows better in the absence of cycloheximide regardless of the temperature (28 °C, 37 °C). By IF, the inclusion morphology at 28 °C is heterogenous and inclusions tend to grow around host cell nuclei. The ultrastructural features of *C*. *poikilotherma* display EBs and RBs replicating by binary fission.

*C*. *poikilotherma* is susceptible to tetracycline and moxifloxacin but behaves intermediate to resistant to azithromycin (ranging from 2–4 μg/mL). The type strain S15-834K^T^ (DSM 106149, CSUR Q6003) and reference strain S15-834C (DSM 106150, CSUR Q6004) have been deposited at the DSMZ (Deutsche Sammlung von Mikrorganismen und Zellkulturen GmbH, Braunschweig, Germany) and at CSUR (Collection de Souches de l′ Unité Rickettsies WDCM 875, Marseille, France).

