# Author Correction: Novel *Chlamydia* species isolated from snakes are temperature-sensitive and exhibit decreased susceptibility to azithromycin

**DOI:** 10.1038/s41598-022-21033-6

**Published:** 2022-09-28

**Authors:** Eveline Staub, Hanna Marti, Roberta Biondi, Aurora Levi, Manuela Donati, Cory Ann Leonard, Serej D. Ley, Trestan Pillonel, Gilbert Greub, Helena M. B. Seth-Smith, Nicole Borel

**Affiliations:** 1 Institute for Veterinary Pathology, Vetsuisse Faculty, University of Zurich, Winterthurerstrasse 268, CH-8057 Zurich, Switzerland; 2 DIMES, Microbiology, Policlinico S. Orsola, University of Bologna, 40138 Bologna, Italy; 3 Institute of Microbiology, University of Lausanne, CH-1011 Lausanne, Switzerland; 4 Applied Microbiology Research, Department of Biomedicine, University of Basel, Basel, Switzerland; 5 Present Address: SA MRC Centre for Tuberculosis Research, DST/NRF Centre of Excellence for Biomedical Tuberculosis Research, Division of Molecular Biology and Human Genetics, Stellenbosch University, Stellenbosch, South Africa; 6 Present Address: Clinical Microbiology, University Hospital Basel, Basel, Switzerland

Correction to: *Scientific Reports* 10.1038/s41598-018-23897-z, published online 04 April 2018

The Author Correction published on 8^th^ September 2021 contains an error in one of the accession numbers for the type strain H15-1957-10C^T^.

“CSUR Q5983”

should read:

“CSUR Q5984”

