# Supplementary material for: Novel Chlamydia species isolated from snakes are temperature-sensitive and exhibit decreased susceptibility to azithromycin
Source: Sci Rep. 2018 Apr 4;8:5660. doi: 10.1038/s41598-018-23897-z (PMC5884828; doi:10.1038/s41598-018-23897-z)
Supplement: Supplementary file 1 — Supplementary files [file 41598_2018_23897_MOESM1_ESM.pdf]

Novel *Chlamydia* species isolated from snakes are temperature-sensitive and exhibit decreased susceptibility to azithromycin

Eveline Staub, Hanna Marti, Roberta Biondi, Aurora Levi, Manuela Donati, Cory A. Leonard, Serej D. Ley, Trestan Pillonel, Gilbert Greub, Helena M.B. Seth-Smith, Nicole Borel

Supplementary Figure 1. Growth curves of *C. pneumoniae* K6 and snake isolates at 24, 32, 40 and 48 hours post infection (hpi)

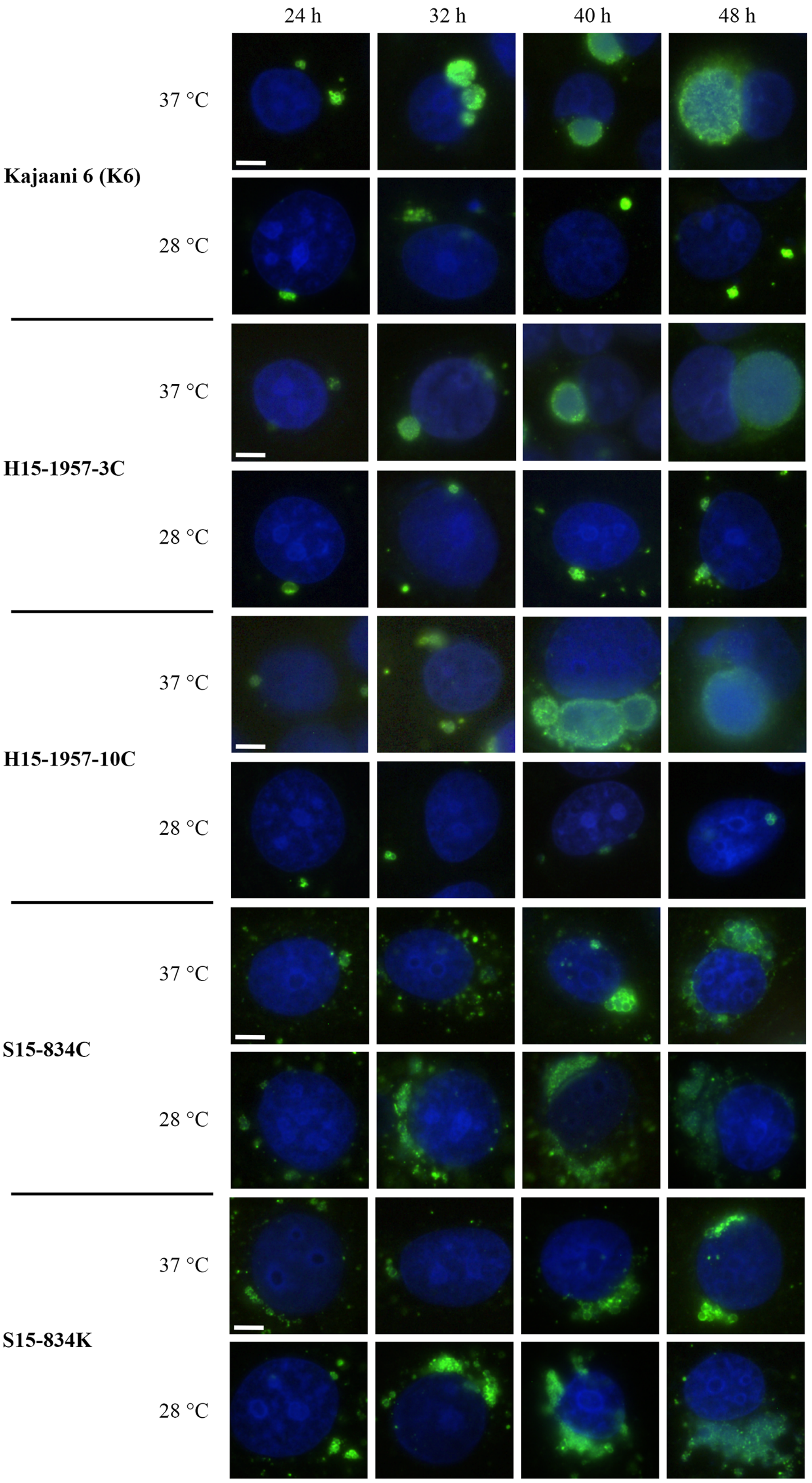

Shown are representative immunofluorescence images illustrating the morphology of *C. pneumoniae* K6, H15-1957-3C, H15-1957-10C, S15-834C and S15-834K (top to bottom) at 24 (column 1), 32 (column 2), 40 (column 3) and 48 (column 4) hpi at 37 °C (top line per strain; lines 1, 3, 5, 7, 9) and 28 °C (bottom lane per strain; lines 2, 4, 6, 8, 10). The size bar indicates 5 µm. Chlamydial inclusions are shown in green, the LLC-MK2 nuclei (DAPI) are shown in blue.

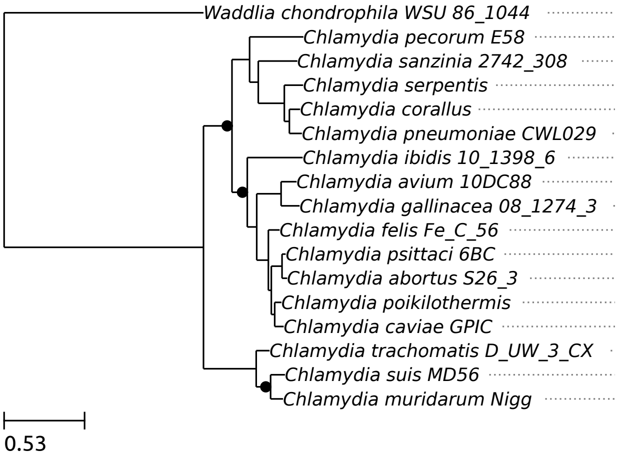

Species	DnaA	SucA	protein_325	FabI	RpoN	FtsK	PepF	Adk	HemL
Waddlia chondrophila WSU 86_1044	45.48	46.48	39.9	68.12	32.07	50.87	43.81	44.71	41.67
Chlamydia pecorum E58	77.34	71.46	68.87	84.23	58.19	73.37	67.6	56.81	57.14
Chlamydia sanzinia 2742_308	76.3	75.83	73.82	84.28	60.05	74.63	68.05	53.7	58.31
Chlamydia serpentis	79.13	74.83	72.17	86.29	60.28	72.72	67.16	54.93	55.66
Chlamydia corallus	79.3	75.22	74.7	86.29	60.86	72.59	69.41	54.93	59.16
Chlamydia pneumoniae CWL029	80.65	74.67	75.41	86.29	60.76	72.97	67.6	56.34	56.84
Chlamydia ibidis 10_1398_6	82.39	77.15	76.12	85.95	59.52	78.43	69.74	50.0	60.19
Chlamydia avium 10DC88	85.65	79.96	79.67	89.3	69.45	83.12	78.13	65.87	65.9
Chlamydia gallinacea 08_1274_3	80.65	79.14	80.66	86.96	68.65	83.19	76.25	66.04	65.44
Chlamydia felis Fe_C_56	93.04	90.97	92.45	94.65	85.78	93.17	83.72	77.46	82.15
Chlamydia psittaci 6BC	93.26	92.62	93.16	89.63	85.82	91.53	86.51	77.0	79.41
Chlamydia abortus S26_3	93.26	91.52	92.69	92.64	84.87	90.55	86.51	76.53	78.03
Chlamydia poikilothermis	100.0	100.0	100.0	100.0	100.0	100.0	100.0	100.0	100.0
Chlamydia caviae GPIC	93.91	92.51	92.22	96.32	91.49	94.41	93.76	82.63	86.73
Chlamydia trachomatis D_UW_3_CX	78.63	68.18	65.95	84.23	49.41	74.74	65.73	48.6	55.82
Chlamydia suis MD56	78.63	68.29	65.24	85.91	52.26	73.46	67.38	48.13	57.72
Chlamydia muridarum Nigg	78.85	69.8	66.43	86.24	51.07	73.66	67.87	48.13	57.72

Figure S2a: Conservation of nine taxonomic markers as compared to *C. poikilothermis* amino acid sequences (in percent identity). DnaA, SucA, protein 325 and FabI are used to delineate species of the same genus (in blue). RpoN, FtsK, PepF, Adk and HemL are used to discriminate species (in red). The low conservation of RpoN, FtsK, PepF, Adk and HemL support the classification of *C. poikilothermis* as a new species of the genus *Chlamydia*.

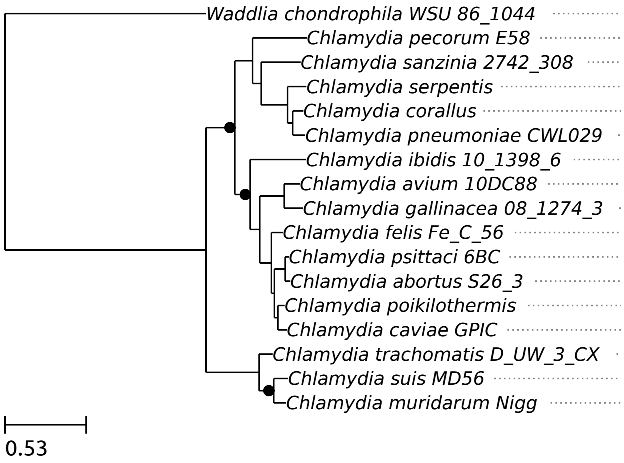

Species	DnaA	SucA	protein_325	FabI	RpoN	FtsK	PepF	Adk	HemL
Waddlia chondrophila WSU 86_1044	47.17	45.71	42.65	68.12	32.46	48.56	42.98	41.43	40.77
Chlamydia pecorum E58	75.82	72.98	67.06	86.58	58.53	72.17	67.92	51.64	58.28
Chlamydia sanzinia 2742_308	77.17	79.38	74.47	84.95	60.76	76.13	69.69	60.85	61.72
Chlamydia serpentis	100.0	100.0	100.0	100.0	100.0	100.0	100.0	100.0	100.0
Chlamydia corallus	88.89	89.32	84.31	93.31	78.4	86.72	81.51	81.69	77.22
Chlamydia pneumoniae CWL029	90.22	88.33	84.07	92.64	76.17	88.04	82.82	77.93	75.63
Chlamydia ibidis 10_1398_6	76.3	71.84	69.65	81.94	56.07	73.05	65.3	50.7	54.91
Chlamydia avium 10DC88	77.17	69.96	69.36	84.62	57.89	72.28	68.09	54.85	59.53
Chlamydia gallinacea 08_1274_3	72.83	71.02	69.95	84.95	56.8	71.17	66.61	54.72	57.91
Chlamydia felis Fe_C_56	80.0	74.06	70.59	87.63	61.37	72.93	67.55	53.99	54.73
Chlamydia psittaci 6BC	81.09	74.94	71.46	84.28	58.63	72.9	66.12	53.05	55.66
Chlamydia abortus S26_3	80.65	73.95	71.56	85.62	58.63	70.65	65.79	53.05	55.89
Chlamydia poikilothermis	79.13	74.83	72.17	86.29	60.28	72.72	67.16	54.93	55.66
Chlamydia caviae GPIC	79.57	74.61	70.59	87.29	60.05	71.96	67.0	55.87	54.73
Chlamydia trachomatis D_UW_3_CX	74.01	65.56	63.98	84.23	50.36	68.22	61.88	45.54	55.11
Chlamydia suis MD56	75.55	65.34	65.16	84.56	47.73	67.31	63.37	45.54	55.34
Chlamydia muridarum Nigg	72.91	66.45	66.27	85.23	47.51	67.78	63.47	48.36	54.87

Figure S2b: Conservation of nine taxonomic markers as compared to *C. serpentis* amino acid sequences (in percent identity). DnaA, SucA, protein 325 and FabI are used to delineate species of the same genus (in blue). RpoN, FtsK, PepF, Adk and HemL are used to discriminate species (in red). The low conservation of RpoN, FtsK, PepF, Adk and HemL support the classification of *C. serpentis* as a new species of the genus *Chlamydia*.

Supplementary Tables

Supplementary Table S1

Sequencing statistics of strains sequenced in this study.

***C.pneumoniae* locus tag**	**Putative function**	***C. serpentis* locus tag**	**Putative function**	**presence in other genome**
CPn_0008-12	hypotheticals			tandem gene duplication
CPn_0013, Cpn_0018-9	pmp			tandem gene duplication
Cpn_0041-47	hypotheticals			absent
Cpn_0049	hypothetical			absent
CPn_0063-4	hypotheticals			absent
CPn_0069	hypothetical			absent
CPn_0124-6	hypotheticals			absent
		C10C_0115	hypothetical	absent
CPn_0142-3	hypotheticals			absent
CPn_0155-6	hypotheticals			absent
		C10C_0138	hypothetical	absent
		C10C_0140 -1	conserved hypothetical, DUF648	tandem gene duplication
		C10C_0144	hypothetical	absent
		C10C_0146	conserved hypothetical, DUF648	tandem gene duplication
		C10C_0148	IncA family	tandem gene duplication
CPn_0173	hypothetical			absent
		C10C_0165	conserved hypothetical, DUF648	absent
CPn_0196 (oppA_2)	Oligopeptide Binding Protein			tandem gene duplication
CPn_0210-5	hypotheticals	*C10C_0183 -7*	IncA family; conserved hypotheticals, DUF648; hypothetical	replacement
CPn_0220-2	hypotheticals			tandem gene duplication
		C10C_0216	hypothetical	tandem gene duplication
CPn_0256-9	hypotheticals			tandem gene duplication
		C10C_0320	conserved hypothetical, DUF1389	tandem gene duplication
CPn_0358	hypothetical			absent
		C10C_0334	hypothetical	c
CPn_0369-70	hypotheticals			tandem gene duplication
		C10C_0337	hypothetical	absent
		C10C_0384	conserved hypothetical, DUF648	absent
CPn_0492-4	hypotheticals			absent
		C10C_0536	hypothetical	replacement

		C10C_0562	hypothetical	replacement
		C10C_0645	hypothetical	tandem gene duplication
CPn_0798	hypothetical			tandem gene duplication
		C10C_0798-9	hypotheticals	tandem gene duplication
CPn_0831	hypothetical			absent
CPn_0881-2	hypotheticals			absent
CPn_0930	hypothetical			absent
CPn_0978	hypothetical			tandem gene duplication
CPn_1054-6	hypotheticals	*C10C_1026-8*	conserved hypotheticals, DUF1978; hypothetical	replacement

Supplementary Table S2

Whole gene differences between the genomes of *C. pneumoniae* CWL029 and *C. serpentis* H15-1957-10C.

Species designation	Strain	Number reads sequence post trimming	Read coverage of chromosome mean ± standard deviation	Read coverage of plasmid mean ± standard deviation
C. serpentis	H15-1947-3C	226964	10 ± 4	108 ± 22
	H15-1957-10C	299978	30 ± 8	47 ± 13
C. poikilothermis	S15-834C	1100343	127 ± 42	201 ± 63
	S15-834K	1253605	118 ± 34	100 ± 29
C. pneumoniae	K6	2023565	50 ± 52	No plasmid

Supplementary Table S3

Finishing primers.

Primer name	Sequence 5'-3'
10C_S7_f2	gaattacgttccccagagca
10C_S7_f1	gcacatgggctatttggttt
10C_S2_f1	tgggctatttggtttggtgc
10C_S7_r1	aagcccagaaaaggtcacag
10C_S7_r2	aattgcgaacaagggtgaag
10C_72_f1	cataacagaggctgcccaat
10C_72_r1	tgtcagtacggcaccttcaa
10C_S2_r1	atgtcagtacggcaccttca
10C_21_f1	aagtagtgctgtgggctgct
10C_21_f2	gtgggctgctgatcacttct
10C_21_r1	cgcatgaatttgccataaaa
10C_21_r2	aacgaaagcaaggccattag
834C_S2_f1	tattgaacgggttggtaggc
834C_S2_r1	gggagcgacattgacagatt
834C_21_f1	caggtggtgctgtggtaatg
834C_21_r1	agagtgcaggggaggagaat
K6_S1_f1	tcctcctctcctcctcccta
K6_S1_r1	tggcaggacaacacaaggta
